# Clinical and Patient Reported Outcomes of an Optimized Trifocal Intraocular Lens

**DOI:** 10.3390/jcm13144133

**Published:** 2024-07-15

**Authors:** Antonio Cano-Ortiz, Álvaro Sánchez-Ventosa, Marta Villalba-González, Timoteo González-Cruces, Juan José Prados-Carmona, Vanesa Díaz-Mesa, David P. Piñero, Alberto Villarrubia-Cuadrado

**Affiliations:** 1 Department of Anterior Segment, Cornea and Refractive Surgery, Hospital Arruzafa, 14012 Cordoba, Spain; alvarosventosa@gmail.com (Á.S.-V.); marta.villalba7@gmail.com (M.V.-G.); timoteogc@gmail.com (T.G.-C.); vdiaz@hospitalarruzafa.com (V.D.-M.); alvillarrubia@yahoo.com (A.V.-C.); 2 Ophthalmology Department, Reina Sofía Hospital, 14004 Cordoba, Spain; 3 Department of Optics, Pharmacology and Anatomy, University of Alicante, 03690 Alicante, Spain

**Keywords:** cataract surgery, spectacle independence, patient-reported outcomes, visual quality, trifocal IOL

## Abstract

**Background/Objectives**: To evaluate the clinical and patient-reported outcomes (PROMs) obtained with an optimized version of a previously investigated trifocal IOL. **Methods**: Prospective non-comparative single-center study enrolling 29 patients (55–71 years) undergoing bilateral cataract surgery with implantation of the trifocal diffractive IOL Liberty 677CMY (Medicontur Medical Engineering Ltd., Zsámbék, Hungary). Visual and refractive outcomes as well as PROMs were evaluated during a 3-month follow-up: measurement of uncorrected and corrected distance (UDVA, CDVA), intermediate (UIVA, DCIVA) and near visual acuities (UNVA, DCNVA), defocus curve, patient satisfaction, photic phenomena perception, spectacle independence, and difficulty in performing some vision-related activities. **Results**: A total of 100%, 92%, and 80% of patients achieved a postoperative binocular UDVA, UIVA, and UNVA of 20/25 or better, respectively. Likewise, 100%, 80%, and 84% of patients achieved a postoperative binocular CDVA, DCIVA, and DCNVA of 20/25 or better, respectively. In the defocus curve, all mean visual acuity values were better than 0.15 logMAR for all defocus levels. A total of 95.8%, 95.8%, and 91.7% of patients referred to be satisfied with their distance, intermediate, and near visual vision, respectively. Mean overall Catquest Rasch calibrated score was −3.12 ± 0.98. Most of the patients were spectacle independent: far (95.8%), intermediate (95.8%) or near vision (91.7%). No bothersome or minimal to moderately bothersome halo, starburst, and glare was perceived by 83.3%, 83.4%, and 83.3% of patients, respectively. **Conclusions**: The trifocal IOL evaluated provides a visual acuity improvement, with high levels of spectacle independence, patient satisfaction, and perceived visual quality associated.

## 1. Introduction

Trifocal diffractive intraocular lenses (IOLs) are an efficacious and safe option of visual rehabilitation after cataract surgery [1] and for presbyopia correction [2]. Several trials have shown that this type of implant provides good levels of distance, intermediate, and near visual acuity [1,2], with high levels of associated spectacle independence [3]. Currently, there are several commercially available trifocal IOLs, with specific differences in their optical design and in their behavior according to the light conditions [4]. The specific optical performance of some of these models can lead to the indication of some of them in patients with specific characteristics and visual demands [4,5]. For this reason, optical bench studies characterizing the optical performance of trifocal IOLs are needed as well as clinical studies demonstrating how these optical properties are converted into visual capabilities for the patients receiving such implants. It should be considered that the relationship between optical bench and clinical data is complex and not easy to interpret [6].

Trifocal IOLs use a diffractive surface on one side of the lens to distribute light to several diffractive orders. It is often assumed that a lens always produces three dominating diffractive orders directly corresponding to the three main focal distances of clinical interest, far, intermediate and near, but this is not always the case [7]. The design of the diffractive surface will provide a specific optical behavior to the IOL, and consequently, a differential visual performance [4]. For example, the trifocal IOL Acriva Trinova IOL (VSY Biotechnology, Germany) has a sinusoidal design, allowing for the desired diffractive effect with fewer diffractive rings and without sharp corners, with the light almost fully distributed to far vision for pupils below 1.4 mm and BO with more light reaching the intermediate and near foci as the pupil size increases (distribution far/intermediate/near: 41/24/35% for 3 mm and 43/34/24 for 4.5 mm) [4]. The FineVision IOL (PhysIOL, Liège, Belgium) is another trifocal IOL based on the combination of a near add (3.50 D) bifocal diffractive profile with an intermediate add (1.75 D) apodized lens. With this IOL, the light at far increases with an increase in pupil, with less light reaching the intermediate focus (48/19/33% for 3 mm and 64/13/23% for 4.5 mm) [4]. Another design of trifocal IOL is that of the AT LISA tri 839 MP IOL (Carl Zeiss Meditec, Jena, Germany), which is trifocal within an IOL diameter of 4.3 mm, whereas it is bifocal between 4.3 mm and 6 mm (+1.66 D addition) and has a diffraction pattern based on proprietary technology called Smooth Micro Phase [4]. This IOL is less pupil dependent, with only some reduction in the light reaching the far focus with an increasing pupil (41/27/33% for 3 mm and 37/28/35% for 4.5 mm). Concerning the PanOptix TFNT00 IOL (Alcon, Fort Worth, TX, USA), this has a diffractive surface on the central 4.5-mm portion of the optic zone with a design based on proprietary optical technology (Enlighten) to redistribute the focal point at 120 cm (0.83 D) to the distance focal point for amplified performance, with two additional foci: intermediate at 60 cm (1.67 D) and near at 40 cm (2.5 D) [4]. With this IOL, there is a significant pupil dependency to favor far vision for scotopic conditions (48/21/31% for 3 mm and 58/19/22% for 4.5 mm) [4,8]. A more recent design of diffractive IOL is that corresponding to the Tecnis Synergy IOL (Johnson & Johnson Vision, Santa Ana, CA, USA), which uses a proprietary diffractive design that results from the combination of the diffractive technologies of Tecnis multifocal (bifocal diffractive profile) and Tecnis Symfony IOLs (diffractive-based EDOF design), with an unusually high step height close to the center of the lens [9] and energy distributions of 43/20/37% and 43/21/36% for 3 and 4.5-mm apertures, respectively [4]. All of these optical peculiarities are responsible for some differences detected between the designs in terms of the defocus curve between trifocal IOLs [10,11,12]. Among the trifocal IOLs that are currently available, the Liberty 677MY (Medicontur Medical Engineering Ltd., Zsámbék, Hungary) is an IOL with a peculiar design based on the use of only seven diffractive rings in the attempt to limit the number of diffractive steps, and consequently the sources of scatter and potentially the levels of photic phenomena. Some previous studies have investigated some of the clinical outcomes that can be obtained with this type of trifocal IOL [13,14,15,16]. However, a recent optimization of this trifocal IOL was performed with a modification of the elevated phase shift (EPS) technology, which was based to optimize the intermediate visual function. The aim of the current study was to evaluate the clinical and patient-reported outcomes obtained with this optimized version of the Liberty trifocal IOL during a 3-month follow-up.

## 2. Materials and Methods

### 2.1. Patients

This was a prospective non-comparative single-center study enrolling a total of 29 patients undergoing uncomplicated phacoemulsification bilateral cataract surgery with the implantation of the trifocal diffractive IOL Liberty 677CMY (Medicontur Medical Engineering Ltd., Zsámbék, Hungary). Inclusion criteria for the study were patients with both eyes having a corneal astigmatism below 1.00 D, age over 50 years of age with indication of cataract surgery or seeking spectacle independence, and expressing consent for participation in the study. Exclusion criteria included active systemic diseases with the potential of altering the outcome of the study, previous ocular surgery including refractive surgery, dry eye, irregular astigmatism, zonular alterations that may affect IOL position and stability, active ocular disease, and previous diagnosis of retinal pathologies and glaucoma.

Before inclusion in the study, each patient was informed in detail about the nature of the study and signed an informed consent according to the tenets of the Declaration of Helsinki. In addition, the study was approved by the medical ethics committee of Arruzafa Hospital (Córdoba, Spain).

### 2.2. Clinical Protocol

All patients had a complete preoperative examination including the measurement of uncorrected (UDVA) and corrected (CDVA) distance visual acuity, manifest refraction, optical biometry and keratometry (IOLMaster 500, Carl Zeiss Meditec, Jena, Germany), corneal topography (Pentacam, Oculus Optikgeräte GmbH, Wetzlar, Germany), Goldman tonometry, endothelial cell count, slit lamp biomicroscopy, and fundus evaluation. Postoperatively, patients were evaluated at 1 day, 1 week, 1 month, and 3 months after surgery. The last postoperative visit included the following tests: monocular and binocular measurement of UDVA and CDVA, manifest refraction, measurement of monocular and binocular uncorrected (UIVA) and distance-corrected intermediate visual acuity (DCIVA) (measured at 66 cm), measurement of monocular and binocular uncorrected (UNVA) and distance-corrected intermediate visual acuity (DCNVA) (measured at 40 cm), measurement of binocular defocus curve (defocus from +2.00 to −4.00 D), evaluation of patient satisfaction and difficulty in performing some vision-related activities using the validated questionnaire Catquest 9SF [17], and evaluation by means of a self-developed questionnaire used in a previous study by our research group [9] of the level of spectacle independence, patient satisfaction, the frequency and level of bothersomeness associated with the perception of some photic phenomena, and the difficulty in performing several daily tasks. Specifically, the questionnaire included the following items:

- In this last week, how often did you wear glasses or contact lenses for performing activities at far, intermediate, and near distances? (all time; most of time; sometimes; occasionally; never)
- What is your level of satisfaction with your distance, intermediate, and near vision without glasses or contact lenses? (fully satisfied; very satisfied; moderately satisfied; somewhat dissatisfied; completely dissatisfied)
- What is your level of satisfaction with your overall vision without glasses or contact lenses? (fully satisfied; very satisfied; moderately satisfied; somewhat dissatisfied; completely dissatisfied)
- Generally, what is your level of satisfaction with your vision to perform the following tasks? (fully satisfied; very satisfied; moderately satisfied; somewhat dissatisfied; completely dissatisfied):
  ○ To read the menu in a dimly lit restaurant;
  ○ To see objects and read street signs at dusk or at night;
  ○ To view the steps or curbs at sunset or at night;
  ○ To read or view photos on a smartphone or tablet;
  ○ To read car dashboard numbers and gauges.
- In this last week, how often have you experienced halos, starbursts, and glare? (never; rarely; sometimes; often-always)
- In general, how much have the halos, starbursts, and glare bothered you? (never; a little; moderately; a lot-extremely)

### 2.3. Surgery

All surgeries were performed by three experienced surgeons using a standard technique of sutureless microincision phacoemulsification. The first step was the instillation of anesthesia and mydriatic drops, and then the surgery was initiated with the creation of a 2.2 mm corneal incision at the temporal area. After this, manual creation of the capsulorhexis (intended diameter of 5 mm) was performed, and the phacoemulsification process was initiated. Once the capsular bag content had been eliminated, the IOL was inserted into the capsular bag through the main incision using the injector provided by the manufacturer for such purpose. At the end of the surgery, a combination of topical antibiotic and steroids was prescribed to be applied postoperatively four times daily for 1 week.

In all cases, the Barrett Universal II formula was used, with the A constant of 118.90 and a target programmed for emmetropia.

### 2.4. Intraocular Lens

The Liberty 677CMY IOL is a pre-loaded one-piece biconvex aspheric trifocal diffractive IOL that incorporates a diffractive area with a 3.0 mm diameter in the anterior surface with seven diffractive rings providing two adds, 1.75 and 3.5 D. The IOL has an overall diameter of 13.0 mm and an optic diameter of 6.0 mm. The angulation of the haptic is 0° with an asymmetric design with posterior vaulting. It is available in powers from +8.0 D to 35.0 D in 0.5-D increments.

### 2.5. Statistical Analysis

Data analysis was performed using the software SPSS version 22.0 for Windows (SPSS, Chicago, IL, USA). Normality of all data distributions was initially evaluated by means of the Kolmogorov–Smirnov test. A descriptive analysis of all continuous variables was carried out, calculating the average values with their corresponding standard deviations and the ranges of maximum and minimum values. For categorical variables, the frequencies of different conditions or aspects were determined. Differences between preoperative and postoperative visits were assessed using the paired Student *t* test or the Wilcoxon test if the data samples compared were or were not normally distributed, respectively. A *p*-value below 0.05 in the tests performed was considered as representative of statistical significance.

The scores obtained with the Catquest 9SF questionnaire were transformed into Rasch calibrated scores using a 4-Andrich rating scale design for statistical analysis purposes, as previously described by the authors who developed the questionnaire [11]. It should be considered that with this questionnaire, higher scoring values are associated with higher levels of difficulty in undertaking vision-related tasks, and consequently lower levels of patient satisfaction.

## 3. Results

A total of 58 eyes from 29 patients with ages ranging from 55 to 71 years old (mean: 64.4 years; standard deviation, SD: 5.3; median: 64.5 years) were enrolled and evaluated in this study. The sample was distributed in gender as follows: 8 males (27.6%) and 21 females (72.4%). Table 1 shows the distribution of preoperative and postoperative data in the right and left eyes of the sample evaluated. 

### 3.1. Visual Acuity Outcomes

**Figure 1:**
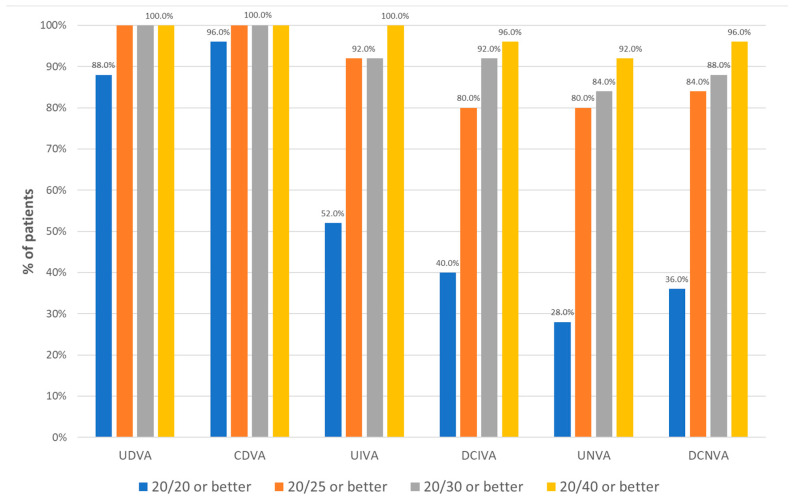
Distribution of postoperative visual acuity data in the sample evaluated. Abbreviations: UDVA, uncorrected distance visual acuity; CDVA, corrected distance visual acuity; UIVA, uncorrected intermediate visual acuity; DCIVA, distance-corrected intermediate visual acuity; UNVA, uncorrected near visual acuity; DCNVA, distance-corrected near visual acuity.

Table 2 summarizes the postoperative monocular and binocular visual outcomes. There was a significant improvement with surgery in UDVA and CDVA (*p* < 0.001). A total of 100%, 92%, and 80% of patients achieved a postoperative binocular UDVA, UIVA, and UNVA of 20/25 or better, respectively (Figure 1). Likewise, a total of 100%, 80% and 84% of patients achieved a postoperative binocular CDVA, DCIVA, and DCNVA of 20/25 or better, respectively (Figure 1). Furthermore, postoperative binocular DCIVA and DCNVA was 20/30 or better in 92% and 88% of patients, respectively (Figure 1).

**Figure 2:**
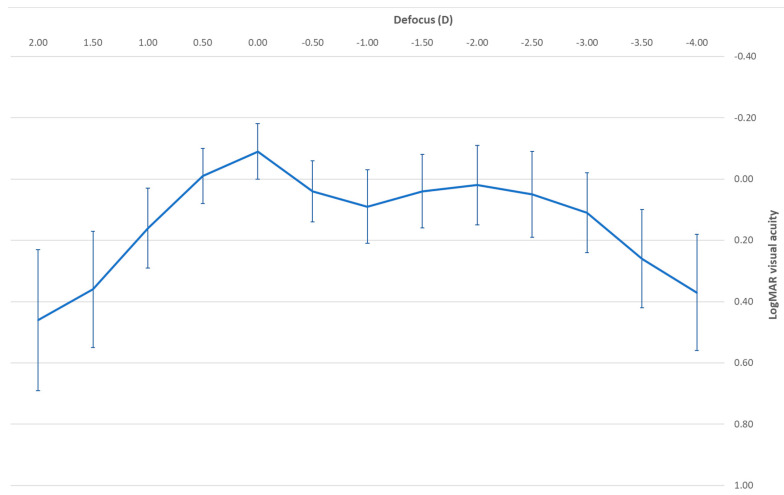
Mean postoperative binocular defocus curve in the sample evaluated.

Figure 2 displays the mean postoperative binocular defocus curve measured in the sample evaluated. As shown, all mean values were better than 0.15 logMAR for all defocus levels evaluated (Figure 2). The corrected-distance visual acuity achieved with the defocus level of −0.50 D did not differ significantly from those measured with the defocus levels of −1.50 (*p* = 0.746), −2.00 (*p* = 0.683), and −2.50 D (*p* = 0.714).

### 3.2. Patient Satisfaction Outcomes

**Figure 3:**
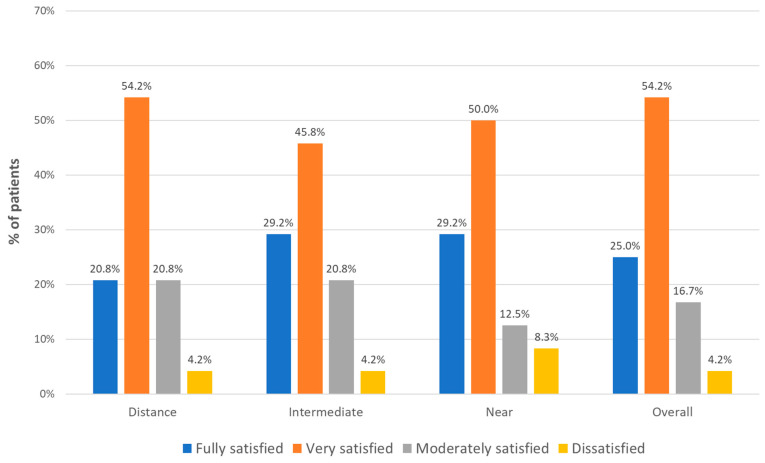
Distribution of patient satisfaction data regarding distance, intermediate, near, and overall vision achieved after surgery.

**Figure 4:**
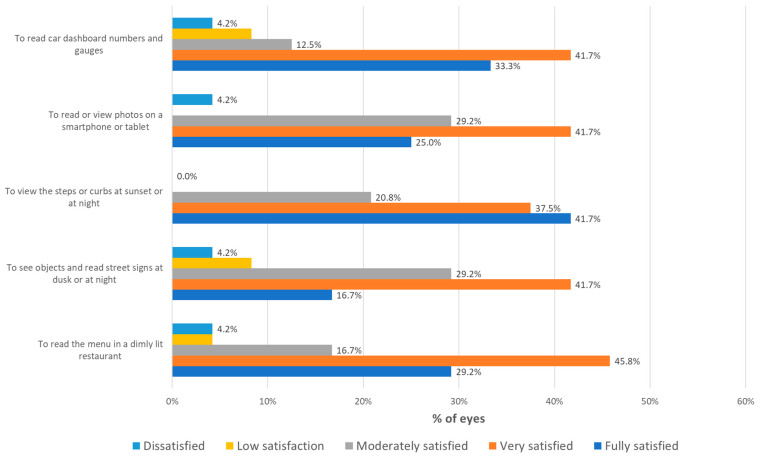
Distribution of the level of patient satisfaction with the vision achieved to perform different daily life activities.

Figure 3 summarizes the results in terms of patient satisfaction with the vision achieved after surgery. As shown, a total of 95.8%, 95.8%, and 91.7% of patients were satisfied with the distance, intermediate, and near visual outcome achieved (Figure 3). In terms of overall vision, a total of 95.8% of patients were satisfied with the outcome obtained (Figure 3). Likewise, a total of 91.7%, 87.6%, 100.0%, 95.9%, and 87.5% of patients were satisfied with the level of vision achieved to read a menu in a dimly lit restaurant, see objects and read street signs at dusk or at night, view steps or curbs at sunset or at night, read or view photos on a smartphone or tablet, and read car dashboard numbers and gauges, respectively (Figure 4).

Table 3 summarizes the postoperative Rasch calibrated scoring obtained with the Catquest-9SF questionnaire in the sample evaluated. The median level of difficulty for most of the vision-related activities evaluated was equal to the lower Rasch calibrated score corresponding to each question, which represented no difficulty. Concerning the variability in the answers provided, it was higher for items B, C1, C6, and C7 (higher value of SD). The mean overall Rasch calibrated score of the questionnaire was −3.12 (SD: 0.98; median −3.40; range −4.08 to −0.73).

### 3.3. Spectacle Independence

**Figure 5:**
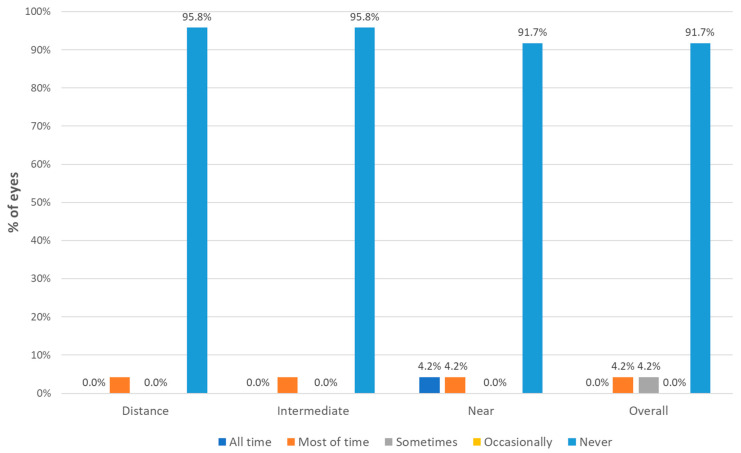
Distribution of the level of spectacle independence achieved at different distances after surgery.

Figure 5 shows the results in terms of postoperative spectacle independence obtained with the trifocal IOL evaluated. Most patients stated that they never needed the use of spectacles after surgery at any distance, far (95.8%), intermediate (95.8%), or near vision (91.7%). In terms of overall vision, the use of spectacles was never needed by 91.7% of patients (Figure 5).

### 3.4. Photic Phenomena

**Figure 6:**
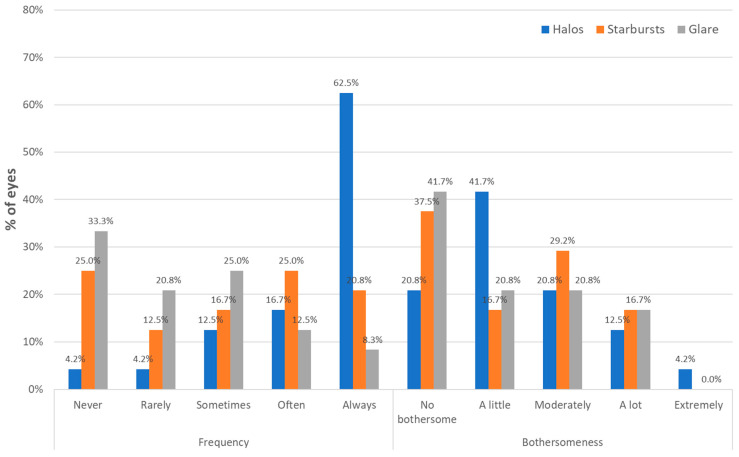
Distribution of the data recorded with the questionnaire concerning the frequency and severity of photic phenomena after surgery.

Figure 6 displays the distribution of the data recorded with the questionnaire concerning the frequency and severity of photic phenomena after surgery. As shown, the most reported photic phenomenon was the perception of halos (62.5%), although the level of bothersomeness for this visual disturbance was minimal or moderate (83.3%) (Figure 6). The perception of starbursts was also usual (always 20.8%, often 25.0%, sometimes 16.7%), but 37.5% described it as not bothersome and minimal to moderate by 45.9% of patients (Figure 6). Finally, glare was not perceived, or perceived rarely or sometimes in 79.1% of patients. This visual symptom was not described as bothersome by 41.7% of patients, and a little or moderately bothersome by an additional 41.6% of patients (Figure 6).

## 4. Discussion

In the current series, a clinical evaluation of the outcomes obtained with an optimized trifocal IOL (Liberty 677CMY), which is based on a slight modification of a previously validated optical design (Liberty 677MY), was performed. This optimization was aimed at enhancing the intermediate visual acuity while maintaining the excellent distance and near visual outcomes of the former model [7,8,9,10]. Indeed, mean postoperative logMAR UDVA values of 0.05 ± 0.08, 0.06 ± 0.12, and −0.04 ± 0.08 were found for the right eyes, left eyes, and binocular conditions, respectively, which is consistent with the results of previous studies [13,14,15,16]. Fernández et al. [13] reported a mean monocular logMAR UDVA value of 0.08 ± 0.11 in a sample evaluating the outcomes of the Liberty 677MY IOL during a 3-month follow-up. Similarly, Cervantes-Coste et al. [14] found a mean 3-month postoperative UDVA value of 0.09 ± 0.09 logMAR in another sample of patients evaluating the same trifocal IOL. Furthermore, our research group [15] reported mean monocular and binocular 1-month postoperative UDVA values of 0.08 ± 0.16 and −0.03 ± 0.13 logMAR, respectively, in a preliminary study evaluating the results of the 677MY IOL model. Concerning CDVA, the outcomes obtained in our series (right eye: −0.03 ± 0.07; left eye: −0.01 ± 0.10; binocular: −0.08 ± 0.06 logMAR) were consistent with those obtained in previous studies evaluating the former model of the trifocal IOL evaluated (Fernández et al. [13]: mono 0.004 ± 0.06; Cervantes-Coste et al. [14]: mono 0.02 ± 0.05; Villarubia Cuadrado et al. [15]: mono 0.01 ± 0.15 and bino −0.07 ± 0.08 logMAR). Aside from agreeing with the data obtained in previous series with the former version of the same trifocal IOL, our distance visual outcomes were comparable to those obtained with other modalities of trifocal IOLs [10,11,12].

Besides the agreement in terms of distance visual outcomes between the 677MY and CMY models), this was also observed for the near visual outcomes. Mean postoperative logMAR UNVA (40 cm) values of 0.14 ± 0.11, 0.15 ± 0.13, and 0.12 ± 0.12 were found for the right eyes, left eyes, and binocular conditions, respectively, which is also consistent with the results of previous studies [14,15,16]. Cervantes-Coste et al. [14] found a mean 3-month postoperative UNVA value of 0.10 ± 0.09 logMAR in their sample of patients implanted with the previous version of the Liberty IOL. The preliminary study of our research group [15] with the 677MY model also provided mean monocular (0.16 ± 0.09) and binocular logMAR UNVA (0.12 ± 0.09) values like those found in the current series. An agreement between the outcomes of the 677MY (monocular: 0.13 ± 0.10; binocular: 0.09 ± 0.09 logMAR) [15] and CMY (monocular: 0.12 ± 0.11; binocular: 0.10 ± 0.11 logMAR) models was also present in terms of DCNVA when compared with previous studies. When compared with other models of trifocal IOLs, some differences could be detected among the trifocal IOLs, although most of them provided similar near visual outcomes [10,11,12,18,19,20,21,22]. Alió et al. [19] studied the visual performance achieved with the PanOptix trifocal IOL from Alcon, obtaining mean logMAR monocular UIVA and UNVA values of 0.12 ± 0.13 and 0.16 ± 0.09, respectively. Galvis et al. [18] evaluated the same trifocal IOL, reporting mean UIVA and UNVA values of 0.07 ± 0.08 and 0.05 ± 0.08 logMAR, respectively, but it should be noted that these visual acuities were measured binocularly. Ribeiro and Ferreiro [11] compared the trifocal IOLs PanOptix and FineVision, obtaining at 3 months after surgery a mean logMAR UNVA of 0.05 ± 0.10 and 0.07 ± 0.11, respectively. In contrast, Mojzis et al. [21] studied the visual performance achieved with the trifocal IOL AT LISA tri from Carl Zeiss Meditec, and they obtained worse logMAR UNVA (0.20 ± 0.10) and DCNVA (0.20 ± 0.10) compared to our data.

In our series, mean postoperative logMAR UIVA (66 cm) values of 0.11 ± 0.10, 0.11 ± 0.09 and 0.06 ± 0.09 were found for the right eyes, left eyes, and binocular conditions, respectively. These results are superior to those reported for the previous version of the Liberty IOL [14,15,16]. Specifically, Cervantes-Coste et al. [14] reported a mean 3-month postoperative UIVA (66 cm) value of 0.18 ± 0.14 logMAR, and Villarubia-Cuadrado et al. [15] obtained mean 1-month postoperative monocular and binocular UIVA (66 cm) values of 0.21 ± 0.16 and 0.17 ± 0.16 logMAR, respectively. This confirms that the optimized model of the Liberty IOL induces an enhancement in the intermediate visual function leading to UIVA and DCIVA values like those reported for other models of trifocal IOLs [10,11,12,18,19,20,21,22]. Ribeiro and Ferreiro [11] found, in their comparison of the trifocal IOLs PanOptix and FineVision, mean 3-month postoperative logMAR UIVA of 0.05 ± 0.08 and 0.11 ± 0.09, respectively. Mojzis et al. [21] found mean 6-month postoperative logMAR values of 0.08 ± 0.09 and 0.08 ± 0.09 for UIVA and DCIVA, respectively, with the trifocal IOL AT LISA Tri. According to this enhanced intermediate vision, an almost flat defocus curve was observed in our series for the optimized trifocal IOL evaluated, with visual acuities better than 0.15 logMAR for all defocus levels, and no significant differences between the corrected-distance visual acuity achieved with the defocus level of −0.50 D and those acuities measured for the defocus of −1.50, −2.00, and −2.50 D. Serdiuk et al. [16] performed a comparative study of three different trifocal IOLs, Liberty 677MY, AT LISA tri 839M, and AcrySof IQ PanOptix, and found similar defocus curves for the three IOLs, although the Liberty IOL seemed to be superior for far and near, while AT LISA tri provided somewhat better visual acuity in the intermediate range.

In agreement with the good results obtained in our series, high levels of patient satisfaction were reported, with 95.8%, 95.8%, and 91.7% of patients satisfied with the distance, intermediate, and near visual outcomes achieved. These values are somewhat better than those reported with the previous model of the Liberty IOL, with 76.9% of patients being fully or very satisfied with their distance, intermediate, and near vision and 92.3% of patients not considering that their level of postoperative vision was leading to some difficulties in the daily life [15]. Indeed, in our series, most patients were satisfied with the level of vision achieved to perform common daily life activities such as reading menus in a dimly lit restaurant, seeing objects and reading street signs at dusk or at night, viewing steps or curbs at sunset or at night, reading or viewing photos on a smartphone or tablet, or reading car dashboard numbers and gauges, respectively. In addition, the patient satisfaction levels obtained with the optimized trifocal IOL were even better than those reported for other models of trifocal diffractive IOLs [23,24,25]. Fernández et al. [25] reported that 78.1% of patients were satisfied with their postoperative spectacle-free vision after the implantation of the AT LISA trifocal diffractive IOL in a sample of presbyopic patients without cataract. Khoramnia et al. [23] compared two models of trifocal IOLs (POD F GF and POD F from FineVision) and found that a high percentage of patients stopped wearing glasses after surgery, with levels of patient satisfaction greater than 80%.

Besides the evaluation of patient satisfaction and postoperative vision-related difficulties with our self-developed questionnaire, an additional evaluation was performed with the validated tool, Catquest 9SF [17]. With this, excellent outcomes were obtained, with the median value of the Rasch calibrated scores representing the difficulty for most of the vision-related activities evaluated as being equal to the score representing no difficulty in each question. The mean overall Rasch calibrated score was −3.12 ± 0.98 (range −4.08 to −0.73), which was consistent with the values reported for other trifocal IOLs [18]. Galvis et al. [18] reported a mean postoperative Rasch-revised Catquest-9SF score of −3.74 ± 0.4 logits (range −3.94 to −2.09) in a sample of eyes implanted with the PanOptix trifocal IOL.

The high level of patient satisfaction observed in the current series was associated with high levels of complete spectacle independence at far (95.8%), intermediate (95.8%), or near vision (91.7%). These spectacle independence rates were better than those reported with the previous model of the Liberty trifocal IOL [15,16]. In their comparative study, Serdiuk et al. [16] found that approximately two-thirds of patients implanted with the trifocal IOLs AT LISA tri and Liberty 677MY could achieve complete spectacle independence, while only 57% of those implanted with the PanOptix IOL could dispose of their glasses. However, Cervantes-Coste et al. [14] reported that nearly all patients (96.4%) included in their sample achieved spectacle independence at all distances after implantation of the Liberty 677MY IOL. Differences in terms of the method used to ask about spectacle dependence, the residual refraction, and patient’s expectancies may account for such differences among studies. It should be considered that the 1-month postoperative spherical equivalent in the study by Cervantes-Coste et al. [14] was −0.23 ± 0.47 D.

Finally, patients were asked about the perception of photic phenomena. There was a perception of photic phenomena, as may be expected with any type of multifocal IOL [26], but the critical point was the level of bothersomeness. The most frequently reported photic phenomena were halos and starbursts (62.5%). In contrast, glare was not perceived, or perceived rarely or sometimes in 79.1% of patients. No patient reported the perception of extremely bothersome starbursts or glare, whereas only 4.2% of patients referred to the perception of extremely bothersome halos. No bothersome or minimal to moderately bothersome halos, starbursts, and glare were perceived by 83.3%, 83.4%, and 83.3% of patients, respectively. This level of photic phenomena is similar to that reported in a previous series evaluating the former model of the Liberty IOL [15,16]. Ribeiro and Ferreiro [11] found in their comparison of the trifocal IOLs PanOptix and FineVision that there were no statistically significant differences found between IOLs in terms of frequency, severity, and bothersomeness of glare, halos, starbursts, hazy vision, blurred vision, distortion, and double vision. The same research group compared the trifocal IOLs PanOptix, Synergy, and FineVision, and showed that no bothersome or minimal to moderately bothersome halos were perceived by 86.7%, 90.0%, and 76.6% of patients implanted with each IOL, respectively. Likewise, these authors confirmed that no bothersome or minimal to moderately bothersome glare was perceived by 83.3%, 83.4%, and 80.0% of patients implanted with each IOL, respectively

This research has some limitations that should be acknowledged. First, no control group was included that would have served as a reference for comparison. Future randomized comparative clinical trials with monofocal, extended depth of focus, or other multifocal IOLs should be performed. Second, the patient-reported outcomes were evaluated by using a self-developed questionnaire that had been used successfully in another study from our research group [9]. However, a consistent evaluation of this questionnaire has not been performed, and this could be considered as a limitation of the study. In any case, the use of this non-validated questionnaire was combined with the use of a consistent validated questionnaire such as the Catquest 9SF, which allowed us to evaluate the impact of the implantation of the trifocal IOL evaluated in different vision-related daily activities. Future studies should be conducted to validate this self-developed questionnaire for the analysis of patient-reported outcomes in cataract surgery. Photic phenomena were also evaluated using this non-validated questionnaire by collecting the subjective perception of the patient. However, the use of an objective method to characterize the photic phenomena is recommended as well as a validated questionnaire to analyze, in a more consistent way, the impact of the photic phenomena generated by the trifocal IOL evaluated. Future trials evaluating the clinical outcomes of this trifocal IOL should consider of all these aspects. Finally, there was a high percentage of females in the evaluated sample, and this can be considered as a limitation because the sample was not equally distributed according to sex. However, to date, no differences in the response to multifocal IOLs have been reported in previous studies as a function of this factor. 

## 5. Conclusions

In conclusion, the implantation of the optimized trifocal IOL evaluated, the Liberty 677 CMY, after cataract surgery provides an enhancement in the distance, intermediate, and near visual acuity, with high levels of patient satisfaction and associated spectacle independence and the perception of minimally bothersome photic phenomena. Specifically, the enhancement in intermediate and near vision achieved with the trifocal IOL led to an almost flat defocus curve, with visual acuities better than 0.15 logMAR for all defocus levels. This was associated with satisfaction rates for the distance, intermediate, and near visual acuity achieved exceeding 90% as well as full distance, intermediate, and near spectacle independence rates of over 90%. The perception of photic phenomena with the IOL evaluated was within the range defined for other trifocal IOLs, with less than 20% of subjects reporting bothersomeness associated with these visual effects. Future comparative studies of this trifocal IOL with other commercially available trifocal IOLs are needed to extract more consistent conclusions about the potential benefit of this implant over the other trifocal IOLs.

## Figures and Tables

**Table 1:** Summary of preoperative data of the sample evaluated. Abbreviations: SD, standard deviation; D, diopters; IOL, intraocular lens; UDVA, uncorrected distance visual acuity; SE, spherical equivalent; CDVA, corrected distance visual acuity; Km, mean keratometry; AXL, axial length; ACD, anterior chamber depth; CCT, central corneal thickness; LT, lens thickness; WTW, white-to-white corneal diameter.

Mean (SD) Median (Range)	Right Eye	Left Eye
LogMAR UDVA	0.24 (0.23) 0.10 (0.00 to 0.70)	0.16 (0.18) 0.10 (0.00 to 0.60)
Sphere (D)	1.03 (2.69) 2.00 (−4.50 to 4.25)	1.32 (2.74) 2.00 (−6.00 to 5.50)
Cylinder (D)	−0.47 (0.44) −0.50 (−1.50 to 0.00)	−0.42 (0.39) −0.50 (−1.50 to 0.00)
SE (D)	0.79 (2.68) 1.38 (−4.50 to 4.00)	1.13 (2.77) 1.75 (−6.00 to 5.50)
LogMAR CDVA	0.13 (0.23) 0.00 (−0.10 to 0.70)	0.09 (0.18) 0.00 (−0.10 to 0.60)
Km (D)	43.67 (1.70) 43.66 (40.60 to 47.34)	43.60 (1.64) 43.49 (40.62 to 46.92)
AXL (mm)	23.16 (1.05) 23.21 (21.34 to 25.78)	23.12 (1.06) 23.06 (21.33 to 25.95)
ACD (mm)	3.02 (0.37) 3.04 (2.47 to 3.76)	3.01 (0.36) 3.03 (2.32 to 3.67)
CCT (µm)	544.38 (26.85) 547.00 (490.00 to 594.00)	546.72 (26.22) 549.00 (496.00 to 590.00)
LT (mm)	4.59 (0.36) 4.57 (3.98 to 5.36)	4.63 (0.32) 4.60 (4.10 to 5.35)
WTW (mm)	11.94 (0.41) 11.90 (11.10 to 12.60)	11.94 (0.39) 12.00 (11.10 to 12.60)
IOL power (D)	22.29 (3.05) 22.50 (15.00 to 29.00)	22.43 (3.37) 22.50 (15.00 to 32.00)

**Table 2:** Summary of the postoperative data of the sample evaluated. Abbreviations: SD, standard deviation; UDVA, uncorrected distance visual acuity; CDVA, corrected distance visual acuity; UIVA, uncorrected intermediate visual acuity; DCIVA, distance-corrected intermediate visual acuity; UNVA, uncorrected near visual acuity; DCNVA, distance-corrected near visual acuity.

Mean (SD) Median (Range)	Right Eye	Left Eye	Binocular
LogMAR UDVA	0.05 (0.08) 0.10 (−0.10 to 0.20)	0.06 (0.12) 0.10 (−0.10 to 0.40)	−0.04 (0.08) 0.00 (−0.20 to 0.10)
LogMAR UIVA	0.11 (0.10) 0.10 (0.00 to 0.30)	0.11 (0.09) 0.10 (0.00 to 0.30)	0.06 (0.09) 0.00 (−0.10 to 0.30)
LogMAR UNVA	0.14 (0.11) 0.10 (0.00 to 0.40)	0.15 (0.13) 0.10 (0.00 to 0.50)	0.12 (0.12) 0.10 (0.00 to 0.40)
LogMAR CDVA	−0.03 (0.07) 0.00 (−0.10 to 0.20)	−0.01 (0.10) 0.00 (−0.10 to 0.40)	−0.08 (0.06) −0.10 (−0.20 to 0.10)
LogMAR DCIVA	0.14 (0.12) 0.10 (0.00 to 0.50)	0.13 (0.12) 0.10 (0.00 to 0.50)	0.09 (0.12) 0.10 (−0.10 to 0.50)
LogMAR DCNVA	0.12 (0.11) 0.10 (0.00 to 0.40)	0.12 (0.11) 0.10 (0.00 to 0.40)	0.10 (0.11) 0.10 (0.00 to 0.40)

**Table 3:** Summary of postoperative Rasch calibrated scoring obtained with the Catquest-9SF questionnaire in the sample evaluated in the current study.

Items	Mean (SD) Median (Range)
Item A: Do you experience that your present vision gives you difficulties in any way in your daily life?	−3.07 (1.31) −3.98 (−3.98 to −1.26)
Item B: Are you satisfied or dissatisfied with your present vision?	−0.73 (1.69) 0.20 (−2.53 to 2.67)
Item C1: Do you have difficulty with reading text in a newspaper because of your vision?	−3.43 (1.72) −4.18 (−4.18 to 3.02)
Item C2: Do you have difficulty with recognizing the faces of people you meet because of your vision?	−3.40 (0.77) −3.63 (−3.63 to −0.91)
Item C3: Do you have difficulty with seeing the prices of goods when shopping because of your vision?	−3.87 (1.13) −4.44 (−4.44 to −1.72)
Item C4: Do you have difficulty with seeing when walking on uneven ground because of your vision?	−3.54 (0.77) −3.77 (−3.77 to −1.05)
Item C5: Do you have difficulty with seeing when carrying out needlework and handicraft because of your vision?	−2.80 (1.14) −3.37 (−3.37 to −0.65)
Item C6: Do you have difficulty with reading text on a television because of your vision?	−3.16 (1.94) −4.59 (−4.59 to 1.98)
Item C7: Do you have difficulty with seeing when carrying out a preferred hobby because of your vision?	−4.08 (1.45) −4.95 (−4.95 to 0.34)

## Data Availability

Data are available on a reasonable request to the authors.
